# Risk prediction models for fear of cancer recurrence in patients with cancer: a systematic review

**DOI:** 10.3389/fpsyt.2026.1940840

**Published:** 2026-10-22

**Authors:** Yifan Zhang, Dongmei Wu, Wendan Xiao, Mao Luo, Lingyan Zhou, Yuanyuan Fan, Baolian Xu, Yanjun Li, Lihong Zhao

**Affiliations:** 1 Department of Radiology, West China Hospital, Sichuan University, Chengdu, Sichuan, China; 2 West China School of Nursing, Sichuan University, Chengdu, Sichuan, China

**Keywords:** cancer, fear of cancer recurrence, prediction model, risk prediction, systematic review

## Abstract

**Background:**

The apprehension of cancer recurrence among cancer survivors strongly influences their quality of life. Predictive models can enable the timely recognition of this concern and the implementation of interventions. In recent years, a multitude of models have been constructed to predict the likelihood of disease recurrence in cancer patients. Nevertheless, the methodological strictness and clinical applicability of these models necessitate further elucidation.

**Objectives:**

Systematic assessment of the existing predictive models for fear of cancer recurrence.

**Methods:**

FOUR databases, including PubMed, the Cochrane Library, Embase, and Web of Science, were systematically searched to identify relevant studies published from the inception of the databases to December 31, 2025. Data extraction was independently performed by two reviewers on the basis of the critical evaluation and data extraction checklist of the systematic review predictive model study. The quality of the included models was assessed using the predictive model bias risk assessment tool.

**Results:**

A total of 1,137 articles were retrieved, from which 15 models across 9 studies were ultimately selected for inclusion. Logistic Regression and machine learning were the most frequently employed methods for model development. The AUC values of the developed models ranged from 0.66 to 0.9786, whereas those reported in validation studies ranged from 0.83 to 0.949. None of the models underwent external validation. All risk prediction models exhibited high bias, primarily because of inappropriate data sources and methodological shortcomings. Social support, age, and education level were identified as common predictive factors.

**Conclusions:**

Current predictive models for the risk of cancer recurrence exhibit good discriminative power. However, most of the included studies are single-center retrospective studies with small sample sizes and lack external validation; these studies exhibit significant methodological limitations that restrict their clinical applicability. Future researchers are encouraged to develop predictive models in accordance with the PROBAST guidelines, enhance methodological rigor, and conduct both internal and external validation.

**Systematic Review Registration:**

https://www.crd.york.ac.uk/PROSPERO/recorddashboard, identifier CRD420261288735.

## 1 Introduction

On a global scale, cancer is among the primary causes of mortality. The International Agency for Research on Cancer reported that in 2020, an estimated 19.3 million new cancer cases and nearly 10 million cancer-related deaths occurred worldwide (1). Although diagnostic and therapeutic technologies have undergone continuous progress in recent years, leading to a marked improvement in patient survival rates, the fear of cancer recurrence (FCR) has surfaced as a pivotal psychological concern for patients during their convalescent period. Fear of cancer recurrence (FCR) is defined as “a patient’s fear, worry, or anxiety about the potential recurrence or progression of cancer” (2). Studies have demonstrated that more than 50% of cancer survivors suffer from a notable fear of cancer recurrence (3).

Various factors contribute to cancer patients’ fear of cancer recurrence, such as a lack of family and social support, advanced tumor staging, poor disease acceptance, and low levels of psychological resilience (4–6). Besides, A high FCR is likely to result in a reduced quality of life (7), worsening depressive conditions (8), and a lack of recovery from disease. Research has indicated that cognitive behavioral therapy can alleviate patients’ fear of cancer recurrence (9). Studies have demonstrated that early intervention can significantly improve patients’ quality of life (10, 11). Therefore, early identification of high-risk populations and prompt intervention are crucial.

Commonly used tools such as the Fear of Cancer Recurrence (FCR) Scale and the Simplified Fear of Disease Progression Scale (FOP-Q-SF) are primarily employed for screening cancer patients who are in the FCR state (12, 13), rather than for identifying high-risk populations; furthermore, the indicators included in these scales are relatively limited. The predictive model can estimate an individual’s risk of experiencing a specific outcome by combining multiple predictive factors (14). This enables early detection and early intervention. Therefore, the development of an effective risk prediction model for the early identification of high-risk individuals and the implementation of personalized interventions are profoundly clinically important for improving the overall prognosis and quality of life for cancer patients. Numerous predictive models have been developed to facilitate the early detection of high-risk FCR patients (15–17). These models are primarily designed for specific patient populations; when developing these models, they incorporate laboratory parameters, pathological staging, demographic characteristics, psychological factors, and other variables, which confers them certain advantages. Nevertheless, extant predictive models display notable heterogeneity in terms of research methodologies, variable selection, and validation performance. Certain models are constructed exclusively on the basis of cross-sectional data and lack prospective validation; others, although they exhibit high discriminative power, are plagued by insufficient calibration or the absence of external validation, thus limiting their clinical applicability.

This study intends to carry out a systematic review to comprehensively search for and analyze published research on predictive models for fear of cancer recurrence; summarize model construction methods, predictive factors, validation strategies, and performance metrics; and assess their methodological quality and risk of bias. The findings offer evidence-based perspectives for optimizing model design and facilitating clinical translation, ultimately contributing to the improvement of psychosocial outcomes among cancer survivors.

## 2 Methods

The study protocol has been registered on the international prospective register of systematic reviews (PROSPERO; registration number: 420261288735).

### 2.1 Search strategy

Four databases, including PubMed, the Cochrane Library, Embase, and Web of Science, were systematically searched to identify relevant studies published from the inception of the databases to December 31, 2025. The following keywords were used for the search: (“Neoplasms”, “cancer OR tumor OR neoplasia OR malignancy”) AND (fear of recurrence OR fear of progression OR fear of cancer recurrence OR FCR) AND (prediction model OR risk prediction OR risk prediction model OR risk model OR nomogram OR risk score OR algorithm OR validation OR external validation OR ROC OR AUC OR c-statistic OR discrimination OR calibration). In addition, we also examined the references of the retrieved studies and related articles to identify any potentially relevant research. The complete electronic search strategy is provided in **Supplementary Table 1**. Besides, we utilized the PICOS system, as recommended by the Checklist for Critical Appraisal and Data Extraction for Systematic Reviews of Prediction Modelling Studies (CHARMS) (18), to provide a comprehensive description of the study population, intervention, comparator, outcome and setting in this review. The key items of this systematic review are described below:

P (Population): Cancer patients concerned about disease recurrence
I (Intervention): Predictive modeling study of fear of cancer recurrence
C (Comparator): Not applicable.
O (outcome): Feeling anxious about the possibility of cancer recurrence
S (Setting): Inpatient and (or) outpatient settings.

### 2.2 Inclusion and exclusion criteria

The following studies meet the following criteria: (1) the researchers are cancer patients; (2) the research design includes only prospective or retrospective cohort studies and cross-sectional studies; (3) the research focuses on constructing, validating, or updating predictive models for assessing the risk of recurrence fear in cancer patients; (4) the outcome of concern is fear of recurrence, and the exclusion criteria are as follows: (1) studies that report only risk factors without constructing predictive models; (2) research on predictive models with fewer than two variables; (3) research not published in English; (4) inability to obtain the full text of the research; and (5) systematic reviews, meta-analyses, conference abstracts and case reports, and duplicate publications.

### 2.3 Study selection

Two researchers independently screened the eligible literature, extracted the data, and cross-checked the data. If there was a disagreement between the two authors on individual literature, a third author was added, and the three authors (Zhang, Wu, and Xiao) discussed and discussed the results together. First, the retrieved records were imported into EndNote X9 software, and duplicates were removed. Afterward, irrelevant articles were excluded by reading the literature titles and abstracts, and a full-text review was conducted for final inclusion. In addition, citation tracking was conducted on all included studies, and reference checks were performed on all included studies.

### 2.4 Data extraction

The data were independently extracted by two researchers using a standardized table based on CHARMS and cross-checked by independent researchers (18). The following two types of data were extracted: (1) basic information: author, year of publication, country, study design, data source, settings, participant characteristics, outcome, outcome measurement, sample size, number of events, and candidate predictors; (2) model information: methods for handling missing data, methods for processing continuous variables, methods for selecting predictors, methods for model development, methods for model validation, model discrimination and calibration, final predictors in the model, and formats of model presentation.

### 2.5 Risk of bias and application assessment

Two researchers used the Predictive Model Bias Risk Assessment Tool (PROBAST) to evaluate the risk of bias and the application of each included study (14). This tool includes four key areas (participants, predictor variables, outcomes, and analysis) and 20 questions to assess the risk of bias in the included studies. The first three domains are used to evaluate the application of the model. Evaluators answer these questions on the basis of their judgment. When all the domains are considered low risk, the overall risk of bias is considered low risk, and when one or more domains are considered high risk, the risk of bias is considered high risk. Any disagreements were resolved through discussion or consultation with a third researcher.

### 2.6 Data synthesis

Given the significant variations in participant characteristics, predictors, and modeling approaches across the included studies, we present the results using a narrative synthesis method. We summarize the information from the included models—such as participant characteristics, outcome measures, approaches to handling missing data and continuous variables, model development and validation methods, model discrimination and calibration results, and model presentation methods.

## 3 Results

### 3.1 Study selection

The flowchart of the research screening process using the systematic review and meta-analysis report project (PRISMA) 2020 is shown in **Figure 1**. A total of 1,137 articles were retrieved from the database, and 261 duplicate articles were excluded. A total of 864 articles were excluded after the titles or abstracts were read, resulting in 12 articles for which the full texts of two could not be obtained. One article was withdrawn, and ultimately, nine articles were included (15–17, 19–24).

**Figure 1:**
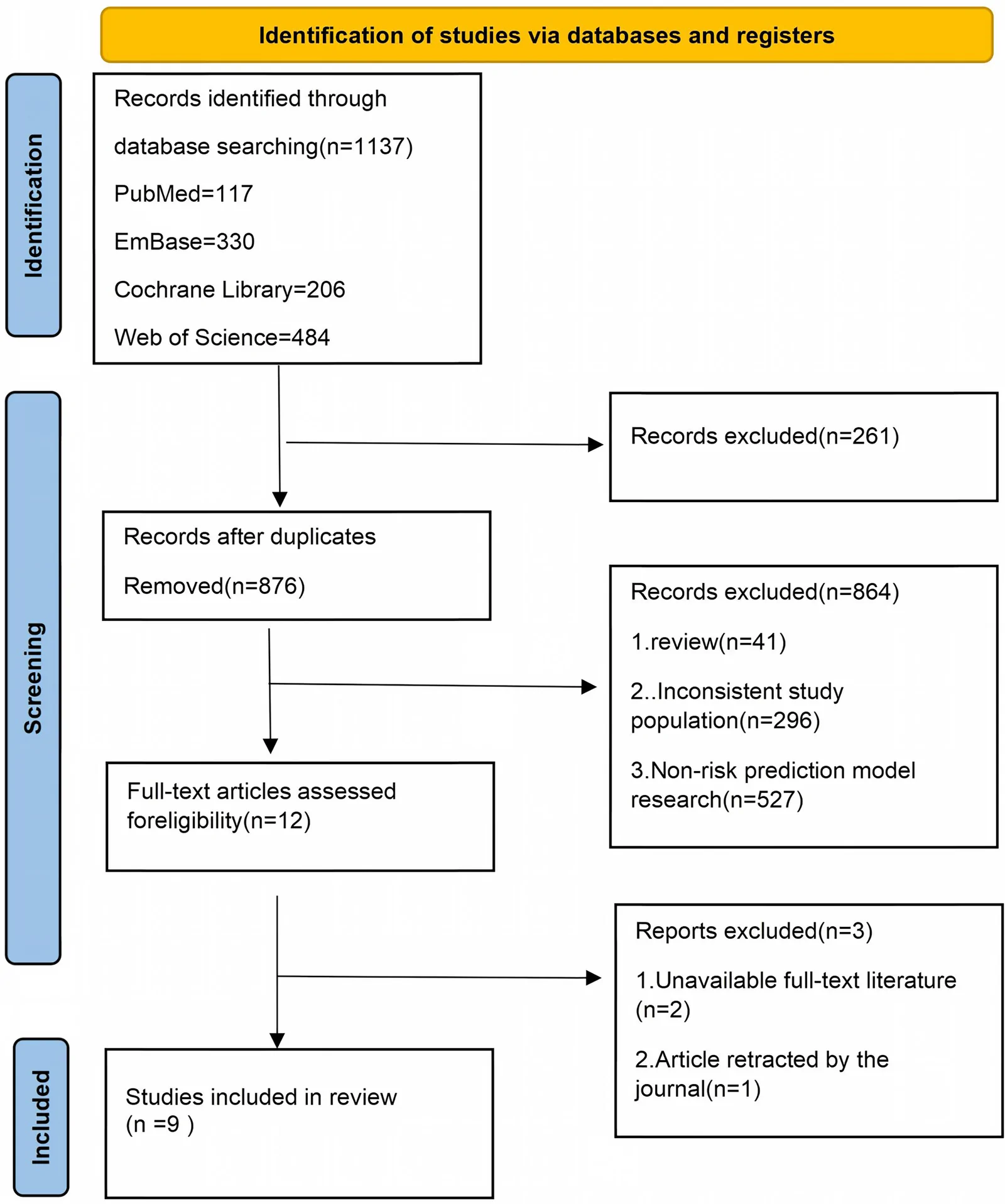
Flow diagram of study selection.

### 3.2 Characteristics of included in the study

**Table 1** shows 15 models from 9 studies published between 2024 and 2025, including cancer patients from China (n = 8) (15–17, 19, 21–24) and France (n = 1) (20). Thirteen models were developed on the basis of cross-sectional data. The number of participants in model development ranged from 202 to 918. The most commonly used assessment tools were the Simplified Fear of Disease Progression Scale (FOP-Q-SF) (n=4), the Simplified Cancer Recurrence Fear Scale (n=4), and the Cancer Recurrence Fear Scale (n=1).

**Table 1:** Characteristics of included studies.

Authors (year)	Country	Study design	Positive	Sample size	Proportion	Participants	Data source	Outcome measures	No. of model
Liu et al., 2024 (24)	China	Cross-sectional	Not reported	596	Not reported	Non-small cell lung cancer	Cancer Hospital of the Chinese Academy of Medical Sciences, (September 2023 and December 2023)	FoP-Q-SF	2
Fei et al., 2025 (23)	China	Cross-sectional	75	202	37.13	Breast cancer patients	A tertiary Grade A hospital in Jiangsu Province	FoP-Q-SF, FCRI-SF	1
Jia et al., 2025 (19)	China	Cross-sectional	174	310	56.13	postoperative cervical cancer patients	Gynecologic Oncology Department of the First Affiliated Hospital of Bengbu Medical University (May 2024 to December 2024).	FoP-Q-SF	1
Koume et al., 2025 (20)	French	Retrospective Cohort	582	918	63.40	Female with no recurrence of breast cancer 5 years after diagnosis patient	healthcare reimbursement data from the French nationwide VICAN survey	FCRI-SF	2
Zhu et al., 2025 (22)	China	Cross-sectional	168	264	63.64	Elderly gastric cancer	First Affiliated Hospital of Anhui Medical University (June 2021 to January 2024)	FoP-Q-SF	1
Ren et al., 2024 (15)	China	Cross-sectional	459	781	58.77	breast cancer	Four Grade-A general hospitals	FCRI-SF	2
Li et al., 2025 (21)	China	Cross-sectional	272	570	47.72	digestive tract tumor (gastric, colorectal and esophageal cancers)	a local hospital (May 2023 to December 2023)	FCRI-SF	1
Wu et al., 2025 (16)	China	Cross-sectional	185	314	58.92	colorectal cancer patients	Fudan University Shanghai Cancer Center (November 2023 and May 2024)	FCRI	2
Xiong et al., 2025 (17)	China	Cross-sectional	174	330	52.73	Patients with gynecological malignant tumors (cervical cancer, ovarian cancer, endometrial cancer, etc.)	A tertiary inpatient patient from China	FoP-Q-SF	3

FOP-Q-SF, Simplified Fear of Disease Progression Scale; FCRI, Cancer Recurrence Fear Scale; FCRI-SF, Simplified Cancer Recurrence Fear Scale.

### 3.3 Characteristics of model development

**Table 2** | details the predictive model for the fear of cancer recurrence among cancer survivors. With respect to the candidate predictors, all the studies employed multiple factors, including sociodemographic characteristics, disease-related factors, and psychological factors; three additional studies incorporated laboratory parameters (17, 19, 20). The number of candidate predictors ranged from 11 to 29, with one study not specifying this parameter count (20). The final predictors, ranging from three to eight components, are presented in **Table 3**, with social support, age, and education level being common predictors. Other predictive factors include tumor size, gender, and others. With respect to the handling of missing values, four studies included only cases with complete data (15, 19, 21, 24),while the remaining studies did not clarify their approaches. Seven studies categorized continuous variables (15, 16, 19, 21–24). In terms of model development, four studies selected final predictors using multivariate logistic regression (19, 21–23), whereas five employed machine learning methods—including random forests, Bayesian hyperparameter optimization, support vector machines, and gradient boosting (15–17, 20, 24).

**Table 2:** Characteristics of risk prediction models for fear of cancer recurrence.

Authors (year)	Model	AUC (development)	AUC (validation)	Candidate predictors	Continuous variable processing method	Missing data	Modeling method	Calibration	Other indexes	model validation	Model presentation
Liu et al., 2024 (24)		LR: 0.726 (0.676–0.775) BPNN: 0.823 (0.782–0.865)	LR: 0.711 (0.630–0.792) BPNN: 0.843 (0.778–0.907)	18 (demographic and clinical data)	Categorical variables	Excluding	LR and BPNN	Not reported	Not reported	internal verification	Not reported
Fei et al., 2025 (23)		0.86 (0.807-0.912)	0.83	15 (Demographics, disease-related variables, psychosocial variables)	Not reported	Not reported	LR	H-L test P = 0.841	Se:0.853 Sp:0.764	internal verification	Equation and Nomogram
Jia et al., 2025 (19)		0.91 (0.853-0.966)	0.909	22 (general information of patients, laboratory indicators and )	Not report	complete data for all analyzed variables	LR	H-L test P = 0.610	Se:0.875 Sp:0.812	internal verification	Equation and Nomogram
Koume et al., 2025 (20)		0.66	Not reported	Not reported	Not reported	Not reported	GB and RF	Not reported	Se:0.7 Sp:0.71	Not reported	Not reported
Zhu et al., 2025 (22)		0.82 (0.77-0.87)	Not reported	11 (Demographics, disease-related variables, psychosocial variables)	Not reported	Not reported	LR	calibration curve and H-L test (p=0.772)	Se:0.77 Sp:0.7	Not reported	nomogram
Ren et al., 2024 (15)		0.937 (0.918-0.957)	0.949 (0.926-0.976)	29 (demographic, clinical characteristics, somatic, cognitive, psychological, social and economic factors, )	Categorical variables	eliminating invalid questionnaires	LR and RF	Calibration curves	Not reported	internal verification	Nomogram
Li et al., 2025 (21)		0.976 (0.966-0.985)	Not reported	24 (FCR, demographics, socioeconomic, clinical characteristics, psychological status and social support)	Categorical variables	questionnaires Those that did not meet the standard were excluded.	LR	H-L test 、calibra tion curve and decision curve	Not reported	internal verification.	Nomogram
Wu et al., 2025 (16)		Not reported	Not reported	23 (sociodemographic factors, disease, and clinical-related variables )	Not reported	Not reported	LR and RF	Not reported	Not reported	Not reported	Not reported
Xiong et al., 2025 (17)											
	LASSO	0.933 (0.9-0.967)	0.898 (0.828-0.969)	20 (Demographics, clinical parameters, psychosocial factors)	Continuous	Not reported	LR	Not reported	F1 score: 0.891	internal verification	Nomogram
	SVM	0.92 (0.883-0.958)	0.889 (0.816-0.962)	20 (Demographics, clinical parameters, psychosocial factors)	Continuous	Not reported	SVM	Not reported	F1 score: 0.881	internal verification	Not reported
	RF	0.968 (0.946-0.987)	0.928 (0.856-0.965)	20 (Demographics, clinical parameters, psychosocial factors)	Continuous	Not reported	RF	Not reported	F1 score: 0.91	internal verification	Not reported

H-L test, Hosmer–Lemeshow test; LASSO, least absolute shrinkage and selection operator-based logistic regression; LR, Logistic regression; AUC, Area under the receiver operating characteristic curve; CI, Confidence interval; RF, Random Forest; SVM, Support Vector Machine; BPNN, Back Propagation Neural Network; GB, Gradient Boosting; SE, Sensitivity; SP, Specificity.

**Table 3:** The final predictive factor in the risk prediction model.

Authors (year)	No. of predictors	The final predictive factor in the risk prediction model for recurrence fear in cancer patients
Liu et al. (2024) (24)	8	Gender, Age, Levels of education, Residence, Monthly income, Whether children are underage, Hope index, Social support
Fei et al. (2025) (23)	4	Education level, surgical approach, quality of life score, cancer recurrence fear score
Jia et al. (2025) (19)	6	Serum albumin level, education level, treatment modality, monthly family income, postoperative complications, SSRS (Social Support Rating Scale)
Koumeet et al. (2025) (20)	4	Drug use, frequency of biological tests, medical procedures, age
Zhu et al. (2025) (22)	8	Age, educational level, disease duration, tumor diameter, postoperative complications, psychological resilience, social support, self-perceived burden
Ren et al. (2024) (15)	5	Fatigue, social constraints, maladaptive cognitive emotion regulation strategies, meta-cognition and age
Li et al. (2025) (21)	6	gender, therapy, alimentary tract hemorrhage, pain, depression and social support
Wu et al. (2025) (16)	3	Brief Illness Perception Questionnaire (BIPQ), Cognitive Emotion Regulation Questionnaire (CERQ), Social Constraints Scale-15 (SCS-15).
Xiong et al., 2025 (17)	4	social support, dyadic coping, mindset bias, and elevated tumor markers

### 3.4 Characteristics of the model validation

**Table 2** outlines the specific characteristics of the model validation. Eight studies reported AUC values of the developed models, ranging from 0.66 (20) to 0.9786 (21), and five studies reported validation model AUC values between 0.83 (23) and 0.949 (15). With respect to model calibration, five studies reported calibration performance using methods such as the Hosmer–Lemeshow test and calibration curves (15, 19, 21–23). One study developed a deep forest model demonstrating optimal discrimination, outperforming both traditional machine learning and conventional regression models, with a validation model AUC of 0.928 (95% CI: 0.856–0.965), an F1 score of 0.889, and an accuracy of 0.900 (17). In terms of model validation, three studies did not conduct any validation (16, 20, 22), six studies performed only internal validation (15, 17, 19, 21, 23, 24), with the bootstrap method being the most commonly used approach, and none of the included studies underwent external validation. Four studies reported the sensitivity and specificity of the model (15, 19, 22, 23).One research paper reports the model’s accuracy, precision, recall, and F1 score. One study conducted a Decision Curve Analysis (DCA), which demonstrated favorable clinical applicability. For presentation, two models were displayed using formulas combined with nomogram plots (19, 23), whereas three models were presented solely as nomogram plots (15, 21, 22).

### 3.5 Summary of risk of bias and applicability

The results of the risk of bias and applicability evaluation using PROBAST are summarized in **Table 4**.

**Table 4:** Results of bias and applicability risk assessment according to PROBAST.

Author(s) (year)	Risk of bias				Applicability Assessment			Overall Evaluation	
	Participants	Predictors	Outcome	Analysis	Participants	Predictors	Outcome	Risk of bias	Applicability
Liu et al., 2024 (24)	–	+	+	–	+	+	+	–	+
Fei et al., 2025 (23)	–	+	+	–	+	+	+	–	+
Jia et al., 2025 (19)	–	+	+	–	+	+	+	–	–
Koume et al., 2025 (20)	–	–	–	–	–	–	+	–	–
Zhu et al., 2025 (22)	–	?	?	–	+	+	+	–	+
Ren et al., 2024 (15)	–	+	+	–	+	+	+	–	+
Li et al., 2025 (21)	–	+	+	–	+	+	+	–	+
Xiong et al., 2025 (17)	–	+	+	–	+	+	+	–	–
Wu et al., 2025 (16)	–	+	–	–	+	+	+	–	+

+ indicates low ROB/low concern regarding applicability; − indicates high ROB/high concern regarding applicability; ? indicates unclear ROB/unclear concern regarding applicability.

PROBAST analysis revealed that all nine studies included in this review exhibited high-risk bias in their subject risk assessments. The subjects in these studies were consistently classified as high risk, primarily because they were all cross-sectional or retrospective cohort studies—designs that do not meet PROBAST’s data source requirements. Such study designs may introduce recall biases during data collection, and none employed random sampling methods.

In the risk assessment of predictive factors, one study was classified as high risk (20), one study as unclear risk (22), and seven studies as low risk. The primary reasons were ambiguous definitions of certain predictive factors in the high-risk studies and incomplete reporting of measurement tools and scoring criteria for some predictive factors.

In the outcome risk assessment, two studies were classified as high risk (16, 20), one study as unclear risk (22), and six studies as low risk. The primary reasons for these classifications were that the high-risk studies did not employ a standardized cancer recurrence fear scale; they failed to specify whether blinded methods were used for outcome measurements, making it impossible to determine whether investigators were aware of the predictor information.

In the statistical analysis of risk assessment, all nine studies were classified as high risk, specifically manifested in the following aspects: ① all nine studies employed univariate analysis to identify predictive factors, potentially overlooking interactions among variables; ② five studies did not report methods for handling missing data (16, 17, 20, 22, 23), while four studies included only complete datasets (15, 19, 21, 24); ③ three studies conducted no model validation (16, 20, 24); six performed only internal validation, and none conducted independent external validation; ④ three studies did not specify whether they addressed data complexities such as multicollinearity or nonlinear relationships (16, 20, 22); and ⑤ three studies did not report model calibration methods (16, 20, 24).⑥Two studies reported that the events per variable (EPV) was less than 20 (22, 23).

In the applicability assessment, three studies were classified as having low applicability (17, 19, 20), whereas the remaining six were rated as having high applicability. The primary reasons include the need to retrieve medical records to obtain certain predictive factors and the inclusion of laboratory parameters, which poses significant challenges in data acquisition and results in poor clinical feasibility.

## 4 Discussion

This systematic review included nine studies that reported 15 predictive models for fear of cancer recurrence. Eight studies provided the AUC values for the development model, which ranged from 0.66 to 0.9786, and the AUC values of the validation models ranged between 0.83 and 0.949. Furthermore, the characteristics and predictive performance of these models are summarized, and their bias risk as well as applicability are evaluated. This demonstrates that the current predictive model used to forecast the risk of cancer recurrence possesses strong discriminative power. however, the results of the PROBAST analysis indicated that all the included studies exhibited significant bias risks, primarily related to participant selection and analytical approaches, which may affect the reliability of the findings.

The risk of significant bias in participant selection primarily stems from inappropriate data sources: all nine included studies were cross-sectional or retrospective cohort studies, which are prone to risks such as subject memory bias, incomplete information, and loss of critical predictive factors. According to the PROBAST guidelines, the use of prospective cohort studies, randomized controlled trials (RCTs), or nested case–control designs can effectively increase the credibility of conclusions (25).

Some studies employ a combination of single-factor and multifactor screening methods to identify predictive factors (19, 21–23); however, such approaches may suffer from issues such as incomplete inclusion of predictive factors or the omission of potential predictive factors because of missing data. While these methods offer good clinical interpretability, they may still have certain limitations in regard to handling complex nonlinear relationships and interactions among variables. Research has shown that variable screening using techniques such as LASSO regression can effectively reduce the risk of model overfitting and improve the accuracy of variable selection (26).

In the analytical domain, all nine included studies exhibited a high risk of bias. In model development studies, two studies reported that the events per variable (EPV) was less than 20 (22, 23); a low EPV may lead to model overfitting (14). With respect to missing value handling, five studies did not report methods for managing missing data, whereas four utilized only complete data, potentially introducing bias into the results. The use of multiple imputation is an optimal approach for addressing missing values (27). With the exception of one study (17), all the others categorized continuous variables, which may lead to information loss and unreliable outcomes (28). To minimize biases arising from variable categorization, researchers are advised to perform statistical analyses using raw data; when nonlinear relationships exist between continuous variables and outcomes, statistical methods such as restricted cubic splines can be employed (29, 30). In terms of modeling methods and applications, most studies employ logistic regression (LR) models. Whereas other studies utilize machine learning methods such as random forests, support vector machines, and gradient boosting (XGBoost), each algorithm has unique characteristics. One study compared forest models with LR models and machine learning approaches and demonstrated superior predictive performance; however, these methods also have significant limitations—such as stringent data requirements and poor interpretability (31). Therefore, when a model is selected, it is essential to carefully consider the trade-off between model interpretability and model performance. Additionally, internal validation and external validation are crucial methods for assessing model stability and reliability (32). More than half of the included studies underwent internal validation, but none conducted independent external validation. Decision curves effectively evaluate the clinical applicability of predictive models by directly comparing the net benefits of different models in clinical decision-making (33). However, only one study in this systematic review utilized decision curve analysis (21). Calibration is a key metric for evaluating clinical prediction models, as it reflects the consistency between a model’s predicted probabilities and the actual observed probabilities; the calibration curve is one of the commonly used evaluation methods (32). In this review, five studies conducted calibration assessments using techniques such as the Hosmer–Lemeshow test or calibration curves. However, four studies did not report their calibration results. Although most models demonstrated good discriminative ability when their AUC values exceeded 0.8, the absence of calibration data may increase the risk of model bias and limit a comprehensive evaluation of model performance (33). This suggests that the current model may perform well only within the specific dataset used in the study; its generalizability and reliability across different populations remain unclear, making it insufficient for clinical translation, and it has a significant risk of application bias. Future research should consider the use of the PROBAST checklist to develop more robust predictive models that enhance practicality and reduce bias.

Predictive factors are a crucial component of predictive models and influence their clinical applicability. The fear of cancer recurrence among cancer patients is influenced by multiple factors, including their own psychological state as well as family and societal factors. Therefore, the included studies included multidimensional predictive factors. In this systematic review, social support emerged as the most common predictive factor in the model for predicting cancer patients’ fear of cancer recurrence. Cancer patients often face significant economic burdens and exhibit poor psychological and physiological conditions (34). A systematic review on the relationship between cancer patients’ fear of cancer recurrence and social support in China demonstrated that higher levels of social support are associated with a lower fear of cancer recurrence (35). Strong social support can provide robust economic support and psychological care for cancer patients. Furthermore, our review identified age, education level, and tumor size as important prognostic factors.These predictors provide a relevant reference basis for future research.It enables the timely provision of tailored, individualized, and effective support to affected patients at an early stage. For example, organize relevant sharing sessions by inviting patients who have currently reached the clinical recovery phase to share their experiences. Additionally, clinical experts in relevant fields can be invited to present cutting-edge treatment approaches both internationally and domestically, thereby boosting patients’ confidence in achieving recovery and alleviating their fear of cancer recurrence. However, owing to variations in cancer type and stage, differences in variable modeling approaches, and methodological differences such as the transformation of categorical variables, predictive factors may exhibit significant heterogeneity; therefore, caution should be exercised when generalizing these findings.

## 5 Limitations

This systematic review has several limitations. Only studies published in the English language were included in this review, which may lead to some bias. First, among the included studies, only one was conducted in France; the rest were survey-based studies performed in China, which may introduce geographical variations. Additionally, owing to significant heterogeneity among the included studies—including differences in sample sources, predictive factors used, and modeling methods—we were unable to quantitatively analyze the performance of the prediction models for cancer patients’ fear of recurrence. Furthermore, most of the included studies underwent only internal validation without external validation, limiting their generalizability. The included studies employed two distinct measurement tools—FCR and FOP-Q-SF—which may have compromised the interpretation and comparability of the study findings.

## 6 Conclusions

Current predictive models for the risk of cancer recurrence exhibit good discriminative power. However, according to the PROBAST tool, all the models demonstrated a high risk of bias. Future researchers are encouraged to develop predictive models that adhere to PROBAST guidelines and account for the clinical context to improve study design and methodology, thereby reducing bias and enhancing model generalizability. Furthermore, comprehensive internal and external validation is essential for developing clinically useful, practical, and scientifically valid prediction tools.

## Data Availability

The original contributions presented in the study are included in the article/**Supplementary Material**. Further inquiries can be directed to the corresponding author.
