# Supplementary material for: Risk prediction models for fear of cancer recurrence in patients with cancer: a systematic review
Source: Front Psychiatry. 2026 Oct 22;17:1940840. doi: 10.3389/fpsyt.2026.1940840 (PMC13736776; doi:10.3389/fpsyt.2026.1940840)
Supplement: Supplementary file 1 [file Table1.docx]

**PUBNED**

|  | Actions | Details | Query | Results |
| --- | --- | --- | --- | --- |
| #6 | … |  | Search:( (("Neoplasms"[Mesh])OR(cancer[Title/AbstractOR tumor[Title/Abstract]OR neoplasia[Title/Abstract]OR malignancy[Title/Abstract]))AND(fear of recurrence[Title/Abstract] OR fear of progression[Title/Abstract]OR fear of cancer recurrence[Title/Abstract]OR FCR[Title/Abstract])AND(prediction model[Title/Abstract]OR risk prediction[Title/Abstract]OR risk prediction model[Title/Abstract]OR risk model[Title/Abstract]OR nomogram*[Title/Abstract]OR risk score[Title/Abstract]OR algorithm*[Title/Abstract]OR validation[Title/Abstract]OR external validation[Title/Abstract]OR ROC[Title/Abstract]OR AUC[Title/Abstract]OR c-statistic[Title/Abstract OR discrimination[Title/Abstract]OR calibration[Title/Abstract]) | 117 |
|  |  |  |  |  |
|  |  |  |  |  |
|  |  |  |  |  |
| #5 |  | > | Seach:prediction model[Title/Abstract]OR risk prediction[Title/Abstract]OR risk prediction model[Title/Abstract] OR risk model[Title/Abstract]OR nomogram*[Title/Abstract]OR risk score[Title/Abstract]OR algorithm*[Title/Abstract]OR validation[Title/Abstract]OR external validation[Title/Abstract]OR ROC[Title/Abstract]OR AUC[Title/Abstract]OR c- statistic[Title/Abstract]OR discrimination[Title/Abstract]OR calibration[Title/Abstract] | 1,326,913 |
| #4 |  |  | Search:fear of recurrence[Title/Abstract]OR fear of | 10,306 |
|  |  |  | progression[Title/Abstract]OR fear of cancer recurrence[Title/Abstract]OR FCR[Title/Abstract] |  |
| #3 |  | > | Search:("Neoplasms"[Mesh])OR(cancer[Title/Abstract]OR tumor[Title/Abstract]OR neoplasia [Title/Abstract]OR malignancy[Title/Abstract]) | 5,377,332 |
| #2 |  | > | Search:cancer[Title/Abstract]OR tumor[Title/Abstract]OR neoplasia[Title/Abstract]OR malignancy[Title/Abstract] | 3,593,162 |
| #1 |  | > | Search:"Neoplasms"[Mesh]Sort by:Most Recent | 4,198,989 |

**EMBASE**

| #6 | #3AND #4 AND #5 | 330 |
| --- | --- | --- |
| #5 | 'calibration'/exp OR calibration OR discrimination:ab,ti OR' c- statistic’:ab,ti OR auc:ab,ti OR roc:ab,ti OR 'external validation':ab,ti OR validation:ab,ti OR algorithm:ab,ti OR risk score"ab,ti OR’ nomogram’ ab,ti OR 'risk model’:ab,ti OR 'risk prediction model"ab,ti OR 'risk prediction' ab,ti OR' prediction model’ ab,ti | 1812307 |
| #4 | ‘fear of recurrence’/exp OR ‘fear of recurrence’ OR (‘fear’/exp OR fear)AND of AND (‘recurrence’/exp OR recurrence)OR ‘fear of progression' ab,ti OR ‘fear of cancer  Recurrence 'ab,ti OR fcr:ab,ti | 21426 |
| #3 | #1 OR#2 | 8692318 |
| #2 | 'cancer/exp OR cancer OR tumor ab,ti OR neoplasia.ab,ti OR malignancy ab,ti | 7792942 |
| #1 | 'neoplasm'/exp | 7023914 |

**Web of Science**

| Search | Neoplasms(Topic)or cancer(Topic)or tumor(Topic)or neoplasia(Topic)or malignancy (Topic) | 5,031,076 |
| --- | --- | --- |
| Search | TS=( "prediction model"OR"risk prediction"OR "risk prediction model"OR "risk model"OR nomogram*OR"risk score"OR algorithm*OR validation OR "external validation"OR ROC ORAUC OR"c-statistic"OR discrimination OR calibration )AND TS=(Neoplasms(Topic)or cancer(Topic)or tumor(Topic)or neoplasia(Topic)or malignancy (Topic)AND TS=fear of recurrence(Topic)or fear of progression (Topic)or fear of cancer recurrence(Topic)or FCR(Topic) | 484 |
| Search | TS=(prediction model)OR TS=(risk prediction)OR TS=(risk prediction model)OR TS=(risk model) OR TS=(nomogram*)OR TS=(risk score)OR TS=(algorithm*)OR TS=(validation)OR TS=(external validation)OR TS=(ROC)OR TS=(AUC)OR TS=(c-statistic)OR TS=(discrimination)OR TS= (calibration) | 7,310,921 |
| Search | TS=fear of recurrence(Topic)or fear of progression (Topic)or fear of cancer recurrence(Topic)or FCR (Topic) | 21,392 |

**Cochrane Library**

| #1 | cancer)ti.ab.kw OR(neoplasms)ti.ab.kw OR (tumor):ti.ab.kw OR(malignancy)ti.ab.kw | 279056 |
| --- | --- | --- |
| #2 | MeSH desoriotor:[Neoplasms] explode all trees | 131245 |
| #3 | #1or #2 | 306362 |
| #4 | fear of recurrence).ti,ab.kw OR (fear of progression)ti.ab.kW OR(fear of cancer recurrence)ti.ab.kw OR(FCR)ti.ab.kw | 2438 |
| #5 | prediction model OR risk prediction OR risk prediction model OR risk model OR nomogram”OR risk score OR algorithm R validation OR external validation OR ROC OR AUC ORc-sta6istie OR discrimination OR calibration | 154866 |
| #6 | #3 and #4 and #5 | 206 |
